# Does weight loss improve semen quality and reproductive hormones? results from a cohort of severely obese men

**DOI:** 10.1186/1742-4755-8-24

**Published:** 2011-08-17

**Authors:** Linn Berger Håkonsen, Ane Marie Thulstrup, Anette Skærbech Aggerholm, Jørn Olsen, Jens Peter Bonde, Claus Yding Andersen, Mona Bungum, Emil Hagen Ernst, Mette Lausten Hansen, Erik Hagen Ernst, Cecilia Høst Ramlau-Hansen

**Affiliations:** 1 Danish Ramazzini Center, Department of Occupational Medicine, Aarhus University Hospital, Denmark; 2 Department of Epidemiology, Institute of Public Health, University of Aarhus, Denmark; 3 Department of Occupational and Environmental Medicine, Bispebjerg Hospital, University of Copenhagen, Denmark; 4 Laboratory of Reproductive Biology, University Hospital of Copenhagen, University of Copenhagen, Denmark; 5 Reproductive Medicine Centre, Skanes University Hospital, Malmö, Sweden; 6 Reproductive Laboratory, Institute of Anatomy, University of Aarhus, Denmark; 7 Department of Gynaecology and Obstetrics, Aarhus University Hospital, Denmark

## Abstract

**Background:**

A high body mass index (BMI) has been associated with reduced semen quality and male subfecundity, but no studies following obese men losing weight have yet been published. We examined semen quality and reproductive hormones among morbidly obese men and studied if weight loss improved the reproductive indicators.

**Methods:**

In this pilot cohort study, 43 men with BMI > 33 kg/m^2 ^were followed through a 14 week residential weight loss program. The participants provided semen samples and had blood samples drawn, filled in questionnaires, and had clinical examinations before and after the intervention. Conventional semen characteristics as well as sperm DNA integrity, analysed by the sperm chromatin structure assay (SCSA) were obtained. Serum levels of testosterone, estradiol, sex hormone-binding globulin (SHBG), luteinizing hormone (LH), follicle-stimulating hormone (FSH), anti-Müllerian hormone (AMH) and inhibin B (Inh-B) were measured.

**Results:**

Participants were from 20 to 59 years of age (median = 32) with BMI ranging from 33 to 61 kg/m^2^. At baseline, after adjustment for potential confounders, BMI was inversely associated with sperm concentration (p = 0.02), total sperm count (p = 0.02), sperm morphology (p = 0.04), and motile sperm (p = 0.005) as well as testosterone (p = 0.04) and Inh-B (p = 0.04) and positively associated to estradiol (p < 0.005). The median (range) percentage weight loss after the intervention was 15% (3.5 - 25.4). Weight loss was associated with an increase in total sperm count (p = 0.02), semen volume (p = 0.04), testosterone (p = 0.02), SHBG (p = 0.03) and AMH (p = 0.02). The group with the largest weight loss had a statistically significant increase in total sperm count [193 millions (95% CI: 45; 341)] and normal sperm morphology [4% (95% CI: 1; 7)].

**Conclusion:**

This study found obesity to be associated with poor semen quality and altered reproductive hormonal profile. Weight loss may potentially lead to improvement in semen quality. Whether the improvement is a result of the reduction in body weight per se or improved lifestyles remains unknown.

## Introduction

The prevalence of overweight and obese individuals is increasing globally [1] and concern is rising over the reproductive consequences of male obesity. Male obesity has been linked to subfecundity [2-4] and a dose-response relationship between increasing BMI and subfecundity has been proposed [3]. Furthermore, male obesity has been associated with abnormal semen characteristics [5-14], although results are conflicting [15-21]. The hormonal abnormality [22-24] associated with obesity is likely to play a major role, and although controversial [25-27], previous studies have shown that the endocrine abnormalities may be reversed by weight reduction [28-33].

Several studies have focused on inhibin B (Inh-B) [34-37], and more recently also anti-Müllerian hormone (AMH), both produced almost exclusively by the Sertoli cells, as markers of spermatogenesis [38-40]. Studies have shown Inh-B to be positively associated with fecundability [41], and obesity has been shown to be associated with a decreased level of Inh-B [5,16]. However, results are conflicting [42,43], and studies on the association between obesity and AMH are lacking.

It is unclear to what extent obesity affects a man's reproductive potential. The existing studies on this subject are all cross-sectional, a limited design for deriving causal inferences. There may be a causal link between male obesity and poor semen quality, however, they may also share a common aetiological factor. Longitudinal studies investigating how weight loss affects semen quality are needed to disentangle these two hypotheses, but no such studies have yet been published. In this paper, we present results from a pilot cohort study with prospectively collected data, investigating how obesity and weight loss affect reproductive hormones including AMH and Inh-B, conventional semen characteristics as well as sperm DNA integrity.

## Methods

### Study population and data collection

Data collection took place from April 2006 to April 2009. Men who participated in a residential weight loss program in Ebeltoft, Denmark were recruited to this pilot cohort study. During the data collection period, men over the age of 18, independent of their weight, were invited to participate and a total of 107 men were invited. Forty-four men (41%) accepted the invitation. Out of the 44 participants, 27 men (61%) took part in the follow-up at the end of the weight loss program. We excluded one man diagnosed with Klinefelter's syndrome, and in the analyses of semen characteristics, two men with azoospermia were excluded because azoospermia probably is not caused by obesity alone.

The weight loss program, based on a healthy diet and daily exercise, lasted approximately 14 weeks. Before and after the weight loss program, the participants had blood samples drawn, provided semen samples and had clinical examinations. The clinical examination was performed on site by one investigator and included height- and weight measurements. Blood samples were drawn by a trained technician between 6:45 a.m. and 8:20 a.m. at baseline and between 7:00 a.m. and 10:30 a.m. after the intervention. The blood samples were transported to the hospital laboratory on dry ice, centrifuged and stored at -80°C until analysed. The participants were asked to provide the semen sample by masturbating into a plastic container after at least 48 hours of sexual abstinence. They were instructed to keep the container close to the body, during transportation to the mobile laboratory on the weight loss centre to avoid cooling, and one trained medical laboratory technician performed all initial semen analyses within one hour after collection. Furthermore, before and after the weight loss program, the participants completed questionnaires on their reproductive experience, medical (e.g. history of diseases in the reproductive organs) and lifestyle factors (e.g. smoking status and alcohol consumption) as well as time and date of the preceding ejaculation, and spillage (if any) during semen sample collection. Finally, testis volume was measured by ultrasound of the testes at baseline by a trained person under the supervision of a medical doctor.

The men received no incentives, and participation was conditional on written informed consent. The regional ethics committee approved the study (reg. number 20060039).

### Analyses of serum samples

Serum samples for testosterone, estradiol, follicle-stimulating hormone (FSH) and luteinizing hormone (LH) were analysed at the Department of Clinical Biochemistry, Aarhus University Hospital, Denmark by Avida Centaur (Bayer Healthcare, Leverkusen, Germany). The sex hormone-binding globulin (SHBG) concentrations were determined using IMMULITE (DPC, Koege, Denmark). Serum concentrations of AMH were measured at the Laboratory of Reproductive Biology, University Hospital of Copenhagen, University of Copenhagen, Denmark using specific ELISA kits according to the manufacturer's instructions (DSL-10-14400; Diagnostic System Laboratories Inc., Webster, TX, USA). Detection limit was 0.05 ng/ml and inter- and intra-assay variations were < 10%. Serum concentrations of Inh-B were measured at the Laboratory of Reproductive Biology, University Hospital of Copenhagen, Denmark using a specific ELISA-kit according manufactures instructions (The Oxford Bio-innovation kit; Biotech-IgG, Copenhagen, Denmark).

### Analyses of semen samples

Semen volume was estimated by weight (1 g = 1 mL). Sperm concentration and sperm motility were assessed as described in 'WHO Laboratory Manual for the Examination of Human Semen-Cervical Mucus Interaction' (World Health Organization, 1999). Analysis of 96% of the samples was initiated within one hour after ejaculation, and within this time it has been shown that the sperm motility is stable [44]. Sperm morphology was assessed using the Tygerberg strict criteria [45]. The laboratory took part in the European Society for Human Reproduction and Embryology external quality control (EQC) program and control tests were in accordance with results obtained by expert examiners within the EQC program.

### Sperm chromatin structure assay (SCSA)

After semen analysis, 100 μL of the raw semen sample was frozen at -80°C for later analysis of sperm DNA integrity. Sperm DNA integrity was analysed by the flow cytometric-based sperm chromatin structure assay (SCSA) at the Reproductive Medicine Centre, Skanes University Hospital, Malmö, Sweden. The details of this analysis have previously been described in detail [46,47]. In brief, the SCSA is based on the fact that damaged chromatin denatures when exposed to an acid-detergent, whereas normal double-stranded chromatin remains stable. After blue-light excitation, the SCSA measures the denaturation of sperm DNA with the dye acridine orange, which differentially stains double- and single-stranded nucleic acids. Five thousand cells were analysed by FACSort (Becton Dickinson, San Jose, CA, USA). Analysis of the flow cytometric data was carried out using dedicated software (SCSASoft; SCSA Diagnostics, Brookings, SD, USA.). The percentage of abnormal sperm with detectable DFI (%DFI) was calculated from the DFI frequency histogram. For the flow cytometer set-up and calibration, a reference sample was used from a normal donor ejaculate sample retrieved from the laboratory repository. The intra-laboratory coefficient of variation for DFI analysis was found to be 4.5%. One investigator blinded to the exposure and other co-variates performed the analyses.

### Statistical analyses

In the cross-sectional study, three groups were formed according to BMI at baseline (1: 33.3 to 41.6 kg/m^2^, 2: 41.7 to 46.08 kg/m^2 ^and 3: 46.1 to 60.9 kg/m^2^). In the longitudinal study, we calculated the percentage weight loss and formed three groups according to percentage weight loss (I: 3.5 to 12.1%, II: 12.2 to 17.1% and III: 17.2 to 25.4%).

Outcome variables included reproductive hormones (testosterone, estradiol, FSH, LH SHBG, AMH and Inh-B as well as the calculated the calculated free androgen index (FAI), the free testosterone⁄free estradiol ratio and LH/free testosterone ratio), conventional semen characteristics (semen volume, sperm concentration, total sperm count, sperm motility and sperm morphology) and DFI. In the longitudinal study, the outcome variables included the differences in the parameters mentioned above.

For each of the outcome variables, crude median, 25^th^, and 75^th ^percentiles were calculated. We performed multiple linear regression analyses with BMI and percentage weight loss as the main determinants. Low BMI/percentage weight loss was considered the reference category. When we tested for trend, BMI and percentage weight loss was entered as a continuous explanatory variable.

In the cross-sectional study, data on the semen characteristics, as well as LH, FSH, AMH, Inh-B, the free testosterone/free estradiol ratio, LH/free testosterone ratio and testis volume were transformed logarithmically to obtain an approximate linear distribution of residuals, whereas no transformations were used on data in the longitudinal study. In the longitudinal study, differences in semen characteristics and reproductive hormones from baseline to follow-up were calculated by subtracting the second sample value from the first sample value, thus a positive difference corresponds to a rise in the characteristics from baseline to follow-up.

A priory, we decided which covariates that potentially should be included in the models, and due to the sample size, we based the selection on a 5% change-in-estimate principle [48]. In the cross-sectional study, the following potential confounders were considered for the regression analyses (see table 1): smoking (yes or no), history of diseases in reproductive organs (cryptorchidism, testicular cancer, surgery in urogenital organs, orchitis and chlamydia infection combined into one variable, present, not present or unknown), season of blood- or semen sampling (April to September or October to March) and age at blood- or semen sampling (continuous). For the analyses on semen characteristics, we also considered the period of abstinence time (< 48 hours, 2 - 5 days or > 5 days), spillage at semen sampling (yes or no) and for analysis of motility also minutes from ejaculation to analysis (continuous). Furthermore, for the regression analyses of reproductive hormones we also considered recent fever.

**Table 1:** Semen characteristics and reproductive hormone levels at baseline according to body mass index (BMI)

Parameter	Body mass index (BMI), kg/m^2^			Test for trend*
	
	33.3 - 41.6 (*n *= 14)^#^	41.7 - 46.08 (*n *= 14)^#^	46.1 - 60.9 (*n *= 15)^#^	*P*-value
Sperm concentration (millions/ml)				
Median (p25, 75)	54 (25, 102)	24 (4, 55)	19 (8, 33)	0.03
Adjusted back-transformed median (95% CI)^a, b, d^	18 (3, 111)	4 (1, 28)	5 (1, 39)	0.02
Semen volume (ml)				
Median (p25, 75)	2.9 (2.2, 4.0)	3.5 (2.2, 5.8)	3.3 (2.4, 4.0)	0.92
Adjusted back-transformed median (95% CI)^a, b, c, d, e^	1.7 (0.8, 3.5)	2.6 (1.3, 5.4)	1.7 (0.7, 4.1)	0.74
Total sperm count (millions)				
Median (p25, 75)	209 (62, 230)	93 (11, 204)	46 (22, 76)	0.03
Adjusted back-transformed median (95% CI)^a, e^	70 (32, 156)	31 (11, 90)	23 (9, 56)	0.02
Normal sperm morphology (%)				
Median (p25, 75)	9 (6, 11)	5 (2, 13)	5 (1, 9)	0.28
Adjusted back-transformed median (95% CI)^a, c, d, e^	10 (0, 244)	7 (0, 103)	2 (0, 61)	0.04
Motile sperm (%)				
Median (p25, 75)	73 (64, 77)	57 (43, 71)	55 (40, 67)	0.06
Adjusted back-transformed median (95% CI)^h^	59 (21, 163)	46 (16, 132)	19 (7, 51)	0.005
DNA fragmentation index, DFI (%)				
Median (p25, 75)	10 (7, 18)	16 (12, 32)	18 (12, 23)	0.23
Adjusted back-transformed median (95% CI)^a, b, d, e, f^	9 (4, 19)	12 (6, 25)	10 (4, 24)	0.70
Testosterone (nmol/L)				
Median (p25, 75)	9.2 (7.8, 11.4)	8.0 (6.4, 11.0)	7.0 (6.0, 8.0)	0.005
Adjusted mean (95% CI)^b, d, e, g^	8.7 (5.3, 12.2)	9.1 (6.0, 12.2)	6.3 (2.6, 10.1)	0.04
Estradiol (nmol/L)				
Median (p25, 75)	0.10 (0.09, 0.15)	0.15 (0.14, 0.17)	0.19 (0.16, 0.23)	< 0.005
Adjusted mean (95% CI)^b, d, e, g^	0.11 (0.07, 0.16)	0.13 (0.09, 0.17)	0.18 (0.13, 0.23)	< 0.005
SHBG (nmol/L)				
Median (p25, 75)	18.0 (12.4, 22.7)	17.4 (14.7, 25.0)	22.8 (15.2, 27.5)	0.62
Adjusted mean (95% CI)^b, d, e, g^	20.5 (13.0, 27.9)	21.5 (14.9, 28.1)	24.2 (16.1, 32.3)	0.07
FSH (IU/L)				
Median (p25, 75)	2.8 (2.6, 3.7)	4.5 (2.2, 5.9)	3.2 (2.2, 3.4)	0.36
Adjusted back-transformed median (95% CI)^b, d, e, g^	2.8 (1.7, 4.6)	3.9 (2.5, 6.2)	2.2 (1.3, 3.9)	0.30
LH (IU/L)				
Median (p25, 75)	3.6 (2.9, 4.6)	4.9 (3.7, 6.8)	3.9 (2.8, 5.2)	0.86
Adjusted back-transformed median (95% CI)^b, d, e, g^	3.1 (2.0, 4.8)	4.7 (3.1, 7.0)	2.9 (1.8, 4.8)	0.60
Inhibin B (pg/ml)				
Median (p25, 75)	160 (141, 220)	123 (117, 170)	120 (86, 171)	0.004
Adjusted back-transformed median (95% CI)^d, e^	156 (94, 257)	128 (84, 195)	110 (64, 188)	0.04
AMH (ng/ml)				
Median (p25, 75)	3.6 (3.1, 4.3)	2.9 (1.8, 4.0)	3.3 (2.2, 4.9)	0.60
Adjusted back-transformed median (95% CI)^b, d, e, g^	2.8 (1.7, 4.7)	2.3 (1.5, 3.7)	2.5 (1.4, 4.3)	0.68
Free androgen index (FAI)				
Median (p25, 75)	59.1 (43.2, 75.8)	45.3 (38.9, 62.8)	33.4 (28.7, 44.0)	0.008
Adjusted back-transformed median (95% CI)^b, d, e, g^	55.0 (36.3, 73.6)	46.3 (29.7, 62.8)	28.5 (8.3, 48.7)	< 0.005
Free testosterone/free estradiol ratio				
Median (p25, 75)	95.2 (76.8, 108.4)	56.2 (45.8, 82.8)	35.6 (32.0, 56.1)	< 0.005
Adjusted median (95% CI)^b, d, g^	69.4 (45.7, 105.2)	59.5 (40.0, 88.3)	32.5 (20.8, 51.0)	< 0.005
LH/free testosterone ratio				
Median (p25, 75)	0.07 (0.06, 0.09)	0.10 (0.08, 0.11)	0.10 (0.08, 0.17)	0.005
Adjusted back-transformed median (95% CI)^b, d, e, g^	0.07 (0.04, 0.10)	0.11 (0.07, 0.17)	0.11 (0.06, 0.18)	0.009
Testis volume (ml)				
Median (p25, 75)	13.5 (11.0, 14.0)	10.0 (8.0, 17.5)	12.0 (10.0, 15.0)	0.80
Adjusted back-transformed median (95% CI)^a, d, e^	8.5 (4.0, 18.5)	8.0 (4.0, 16.0)	10.0 (4.0, 24.0)	0.98

p, percentile; CI, confidence interval. *Trends were tested by Spearman's rank correlation test and multiple linear regression analyses with BMI entered as a continuous explanatory variable. #This number of participants (n) relates to the hormone parameters except for AMH. The numbers in the groups for the following variables are: sperm concentration n = 13, n = 14, n = 14; semen volume n = 13, n = 8, n = 9; total sperm count n = 13, n = 7, n = 10; morphology n = 12, n = 14, n = 14; motility n = 13, n = 14, n = 14; DFI n = 11, n = 14, n = 14; testis volume: n = 5, n = 9, n = 7, AMH n = 13, n = 13, n = 15.

The medians are adjusted for the following: abstinence time (a), current smoking (b), season (c), diseases in the reproductive organs (d), age (e), spillage at semen sampling (f) fever (g) and minutes from ejaculation to start of semen analysis (h).

In the longitudinal study, the following potential confounders were considered (see table 2): differences in smoking status (no difference, smoker at the first sample, but not at the second sample or smoker at the second sample, but not at the first sample) and difference in season (no difference in season, September - April at the first sample and March - October at the second sample or March - October at the first sample and September - April at the second sample). In the semen analyses, the differences in spillage (no difference, spillage at the first sample and not at the second sample or spillage at the second sample and not at the first sample) and the differences in abstinence time (days) were additionally considered, and for analysis of motility, the differences in minutes from sampling to analysis. In the statistical analyses on semen volume and total sperm count, the men reporting spillage were excluded from the analyses.

**Table 2:** Differences in semen characteristics and reproductive hormone levels according to weight loss

Adjusted mean (95% CI) differences in semen and hormone levels	Weight loss in percent (%)			Test for trend*
	
	3.5 - 12.1 (n = 10^#^)	12.2 - 17.1 (n = 10^#^)	17.2 - 25.4 (n = 10^#^)	*P*-value
Sperm concentration (millions/ml)^a, c, d^	-11 (-49, 27)	19 (-23, 61)	17 (-24, 58)	0.33
Semen volume (ml)^c^	-1.0 (-2.3, 0.3)	1.5 (-0.4, 3.5)	1.3 (-0.9, 3.4)	0.04
Total sperm count (millions)^a, c^	-41 (-147, 65)	232 (77, 387)	193 (45, 341)	0.02
Normal sperm morphology (%)^a, b, c^	0 (-2, 4)	1 (-3, 4)	4 (1, 7)	0.16
Motile sperm (%)^a, c, d, e^	-2 (-15, 11)	4 (-10, 18)	11 (-3, 25)	0.22
DFI (%)^a, b, c, d^	7 (-2, 17)	-1 (-11, 9)	0 (-10, 10)	0.96
Testosterone (nmol/L)^a, b^	0.7 (-1.1, 2.5)	3.3 (1.4, 5.2)	3.7 (2.0, 5.4)	0.02
Estradiol (nmol/L)	-0.03 (-0.05, 0)	-0.02 (-0.05, 0)	-0.01 (-0.03, 0.01)	0.93
SHBG (nmol/L)^a, b^	1.7 (-2.2, 5.5)	5.0 (1.0, 9.0)	5.0 (1.4, 8.5)	0.03
FSH (iu/L)^a^	0.1 (-0.3, 0.6)	0.3 (-0.3, 0.8)	0.1 (-0.3, 0.6)	0.95
LH (iu/L)^a, b^	0.7 (-0.6, 2.0)	1.2 (-0.1, 2.6)	0.3 (-0.9, 1.5)	0.85
Inhibin B (pg/ml)^a, b^	-30.1 (-51.7, -8.4)	-22.3 (-44.8, 0.2)	-13.6 (-33.6, 6.4)	0.34
AMH (ng/ml)^a, b^	-0.29 (-0.65, 0.07)	-0.02 (-0.42, 0.38)	0.24 (-0.09, 0.59)	0.02
Free androgen index (FAI)^a, b^	-3.7 (-13.3, 6.0)	3.5 (-6.5, 13.6)	6.5 (-2.4, 15.4)	0.43
Free testosterone/free estradiol ratio^a^	15.0 (0.5, 29.4)	38.3 (22.1, 54.4)	25.7 (11.4, 40.0)	0.18

CI, confidence interval. *Trends were tested by multiple regression analyses with weight loss in percent entered as a continuous explanatory variable. #This number of participants (n) relates to the differences in hormone parameters, except for AMH. The numbers in the groups for the following variables are: sperm concentration n = 9, n = 9, n = 9; semen volume n = 7, n = 4, n = 4; total sperm count n = 6, n = 4, n = 4; morphology n = 9, n = 9, n = 9; motility n = 8, n = 9, n = 9, DFI n = 8, n = 9, n = 9 and AMH n = 10, n = 9, n = 10

The means are adjusted for the following: difference in season (a), difference in smoking status (b), difference in abstinence time (c), difference in spillage at semen sampling (d) and difference in minutes from ejaculation to start of semen analysis (e).

We performed sub-analyses to check consistency of our results, using differences in BMI as the explanatory variable instead of weight loss in percent. Finally, due to the low number of participants in the analyses of semen volume and total sperm count after exclusion of participants with spillage, we performed two sub-analyses with all participants included and adjusted for spillage instead. In one model, we adjusted for the covariates by using the difference (e.g. difference in spillage) from baseline to follow up, as described above. Additionally, we fitted a model with total sperm count at follow-up as a function of the weight loss, controlling for total sperm count at baseline as well as the other covariates (spillage, abstinence time and season).

The statistical analyses were performed by using Stata 11 software (Stata Corporation, Cillege Station, TX). A two-tailed *P *value of < 0.05 was considered statistically significant.

## Results

The median (range) age was 32 (20-59) years. The median (range) BMI was 44 (33 - 61) kg/m^2^. In table 1, the semen characteristics and reproductive hormone levels at baseline according to BMI are presented. After adjustment for potential confounders, BMI was inversely associated with sperm concentration, total sperm count, normal sperm morphology, and motile sperm. The group with the highest BMI had a 71% (95% CI: -6; 92) lower sperm concentration and 68% (95% CI: 14; 88) lower total sperm count than the group with the lowest BMI. For semen volume and DFI, no statistically significant trends were observed, however, the median DFI tended to increase with higher levels of BMI. Furthermore, BMI was negatively associated with testosterone and Inh-B and positively associated with estradiol at baseline. The calculated FAI and free testosterone⁄free estradiol ratio were lower at higher levels of BMI. The data indicated a higher level of SHBG with higher levels of BMI, although not statistically significant. There was no difference in testis volume in the groups (Table 1).

Following the weight loss program, the median (range) weight loss was 22 (4; 39) kg, corresponding to a median percentage weight loss on 15%, ranging from 3.5% to 25.4%. In table 2, the adjusted mean (95% CI) differences in semen characteristics and reproductive hormone levels according to weight loss in percent are presented. After adjustment, the percentage weight loss was positively associated with an increase in total sperm count and semen volume. The group with the largest weight loss had a statistically significant increase in both total sperm count [193 millions (95% CI: 45; 341)] and morphology [4% (95% CI: 1; 7)]. We observed no difference in DFI from baseline to follow-up. When using the differences in BMI instead of percentage weight difference as the explanatory variable, the direction and magnitude of the associations were essentially unchanged. Additionally, the percentage weight loss was associated with an increase in testosterone, SHBG and AMH, and FAI and the free testosterone/free estradiol ratio tended to increase with increasing weight loss in percent.

Finally, the results from the sub-analyses with semen volume and total sperm count with all participants were in the same direction, however, attenuated as expected, and p-values were no longer below 0.05.

## Discussion

The study showed that a high BMI at baseline was associated with low values of total sperm count, sperm concentration, normal sperm morphology, and motile sperm. Weight loss was associated with an increase in total sperm count and semen volume among men who participated in a 14-week weight loss program. Additionally, the weight loss was associated with an increase in testosterone, SHBG and AMH, and FAI improved significantly in the group with the largest weight reduction. Weight loss did not decrease serum estradiol levels.

As far as we know, this is the first cohort study investigating the association between weight loss and semen quality. Thus the results are unchallenged and further research is necessary to disclose the matter further. Our results indicate that there is a causal inverse association between BMI and semen quality, and that it may be possible to improve semen quality by a weight reduction. However, we cannot exclude that changes in lifestyle, diet or exercise caused the observed improvement in semen quality, rather than the reduction in weight per se.

Despite conflicting results [15-21], previous studies (all cross-sectional) have mainly shown low sperm concentration among overweight and obese men [5,8,9,11,12,49], similar to what we find. Considering the well-established association between male obesity and altered reproductive hormonal profile, and the fact that testosterone is required in large concentrations to maintain spermatogenesis, it is reasonable to consider obesity to also affect semen quality. Thus we believe that the inverse association between BMI and semen quality is not a chance finding.

The hormonal profile among obese men evaluated in this study was characterized by abnormalities in the sex hormones, and weight loss improved some of the hormone levels, however, they were not normalized. It should be noted that the men were severely obese at baseline and remained overweight or obese after the weight loss program. This could explain why we did not observe a larger improvement in the hormonal parameters. The previous published studies, reporting improvement or normalization of the reproductive hormones, were on less obese men than in this present study.

Inh-B and AMH are produced almost exclusively by the Sertoli cells and have been proposed as markers of spermatogenesis. Inh-B have been found to be significantly lower in men with testicular dysfunction [34-36] and AMH to be significantly lower in subfertile men [38-40]. Therefore, we expected both hormones to be negatively associated with BMI, but this was only seen for Inh-B, as previously reported [16]. In this present study we compared severely obese men, all with BMI above 30 kg/m^2 ^when entering the study and the AMH levels among these men might be lower than normal weight men, which could explain why we see no difference when comparing the two groups with the most obese men with the least obese men. Tüttelmann *et al*. [43] showed that, among men with a median BMI of 25.7 kg/m^2^, the median (range) concentration of AMH was 6.3 ng/mL (1.8; 26.8), higher than among the men in our study where the median (range) AMH concentration was 3.3 ng/mL (0.2; 10.7). Furthermore, we hypothesized that Inh-B and AMH would improve by weight loss but only AMH increased significantly.

The major strength of this study is the successful weight loss program, providing prospectively collected data, which adds new important information to the existing cross-sectional studies. The risk of misclassification of the outcome variables is limited and most likely non-differential, since analyses of semen and blood samples were performed blinded to the exposure variables. Misclassification of the exposure variables is unlikely since anthropometric measurements were obtained on-site by one investigator and do not depend on self-reports. From the questionnaires, data were available on the main factors that we think affect semen quality, such as abstinence time and diseases of the reproductive organs. However, confounding from other unknown factors is possible and our findings may also be due to chance, since the sample size is small.

The major limitation in this study is the limited sample size, resulting in wide confidence intervals, and the results must therefore be interpreted with caution. The participation rate (41%) is low, and leaves open the possibility of selection among participants. However, to cause bias away from the null, selection has to be related to both semen quality and BMI, and the participation rate of men with poor semen quality and high BMI must be higher. We have no reason to suspect participation to be associated with the exposure and the risk of differential participation and selection bias is limited, although it is possible as a chance phenomenon. Furthermore, loss to follow-up leaves room for selection bias, if attrition is dependent on the change in semen quality as well as related to the weight loss. Therefore, we examined if those who dropped out of the study systematically differed from those who remained in the sample. The two groups were found to have similar weight, BMI and reproductive hormones at baseline. Sperm concentration and total sperm count were lower among loss to follow-up men than among those who remained and the direction of this selection bias could be both away and toward the null.

Finally, the follow-up period was on average 103 days (ranging from 86 to 111 days), and spermatogenesis takes approximately 64 days [50]. Thus the follow-up period in the present study should be able to detect changes on the early stages of spermatogenesis, although a longer follow-up period would be desirable.

Thirty-four percent of the men had sperm concentrations below the World Health Organization (2010) referent level of 15 million/ml when entering the study. The median (p25, 75) sperm concentration of all participants at baseline was 25 (12, 64) million/ml and 19 (8, 33) million/ml among the most obese men. Since fecundity increases with sperm concentrations up to approximately 40 million/mL [51], some may have problems fathering a child.

## Conclusions

To conclude on this pilot cohort study, we observed that the altered androgen profile tended to improve following weight loss and that weight loss may potentially lead to improvement in semen quality, although we can not conclude this to be a result of the reduction in body weight per se. The observation has biologic plausibility, but the findings should be replicated in a larger cohort with longer follow-up time including a wider range of BMI levels.

## Competing interests

The authors declare that they have no competing interests.

## Authors' contributions

LBH contributed to analysis and interpretation and drafted the manuscript. AMT contributed to study design, acquisition of data, analysis and interpretation of data. ASA contributed to study design, acquisition of data and interpretation of data. JO contributed to analysis and interpretation of data. JPB contributed to study design and analysis and interpretation of data. CYA contributed to acquisition of data and interpretation of data. MB contributed to acquisition of data and interpretation of the data. EHE contributed to acquisition of data and interpretation of data. MLH contributed to analysis and interpretation of data. EE contributed to study design, acquisition of data and interpretation of data. CHRH contributed to study design, acquisition of data, analysis and interpretation of data. All authors read and approved the final manuscript.

## References

[B1] Haslam DW James WP Obesity Lancet 2005 366 1197 1209 10.1016/S0140-6736(05)67483-1 16198769

[B2] Sallmen M Sandler DP Hoppin JA Blair A Baird DD Reduced fertility among overweight and obese men Epidemiology 2006 17 520 523 10.1097/01.ede.0000229953.76862.e5 16837825

[B3] Ramlau-Hansen CH Thulstrup AM Nohr EA Bonde JP Sorensen TIA Olsen J Subfecundity in overweight and obese couples Hum Reprod 2007 22 1634 1637 10.1093/humrep/dem035 17344224

[B4] Nguyen RH Wilcox AJ Skjaerven R Baird DD Men's body mass index and infertility Hum Reprod 2007 22 2488 2493 10.1093/humrep/dem139 17636282

[B5] Jensen TK Andersson AM Jorgensen N Andersen AG Carlsen E Petersen JH Body mass index in relation to semen quality and reproductive hormones among 1,558 Danish men Fertil Steril 2004 82 863 870 10.1016/j.fertnstert.2004.03.056 15482761

[B6] Fejes I Koloszar S Szollosi J Zavaczki Z Pal A Is semen quality affected by male body fat distribution? Andrologia 2005 37 155 159 10.1111/j.1439-0272.2005.00671.x 16266392

[B7] Magnusdottir EV Thorsteinsson T Thorsteinsdottir S Heimisdottir M Olafsdottir K Persistent organochlorines, sedentary occupation, obesity and human male subfertility Hum Reprod 2005 20 208 215 15567884 10.1093/humrep/deh569

[B8] Koloszar S Fejes I Zavaczki Z Daru J Szollosi J Pal A Effect of body weight on sperm concentration in normozoospermic males Arch Androl 2005 51 299 304 10.1080/01485010590919701 16036638

[B9] Fejes I Koloszar S Zavaczki Z Daru J Szollosi J Pal A Effect of body weight on testosterone/estradiol ratio in oligozoospermic patients Arch Androl 2006 52 97 102 10.1080/01485010500315479 16443585

[B10] Kort HI Massey JB Elsner CW Mitchell-Leef D Shapiro DB Witt MA Impact of body mass index values on sperm quantity and quality J Androl 2006 27 450 452 10.2164/jandrol.05124 16339454

[B11] Hammoud AO Wilde N Gibson M Parks A Carrell DT Meikle AW Male obesity and alteration in sperm parameters Fertil Steril 2008 90 2222 2225 10.1016/j.fertnstert.2007.10.011 18178190

[B12] Stewart TM Liu DY Garrett C Jorgensen N Brown EH Baker HW Associations between andrological measures, hormones and semen quality in fertile Australian men: inverse relationship between obesity and sperm output Hum Reprod 2009 24 1561 1568 10.1093/humrep/dep075 19351657

[B13] Chavarro JE Toth TL Wright DL Meeker JD Hauser R Body mass index in relation to semen quality, sperm DNA integrity, and serum reproductive hormone levels among men attending an infertility clinic Fertil Steril 2010 93 2222 2231 10.1016/j.fertnstert.2009.01.100 19261274 PMC2864498

[B14] Hofny ERM Ali ME bdel-Hafez HZ Kamal EE-D Mohamed EE bd El-Azeem HG Semen parameters and hormonal profile in obese fertile and infertile males Fertil Steril 2010 94 581 584 10.1016/j.fertnstert.2009.03.085 19423100

[B15] Qin DD Yuan W Zhou WJ Cui YQ Wu JQ Gao ES Do reproductive hormones explain the association between body mass index and semen quality? Asian J Androl 2007 9 827 834 10.1111/j.1745-7262.2007.00268.x 17968470

[B16] Aggerholm AS Thulstrup AM Toft G Ramlau-Hansen CH Bonde JP Is overweight a risk factor for reduced semen quality and altered serum sex hormone profile? Fertil Steril 2008 90 619 626 10.1016/j.fertnstert.2007.07.1292 18068160

[B17] Pauli EM Legro RS Demers LM Kunselman AR Dodson WC Lee PA Diminished paternity and gonadal function with increasing obesity in men Fertil Steril 2008 90 346 351 10.1016/j.fertnstert.2007.06.046 18291378 PMC2597471

[B18] Ramlau-Hansen CH Hansen M Jensen CR Olsen J Bonde JP Thulstrup AM Semen quality and reproductive hormones according to birthweight and body mass index in childhood and adult life: two decades of follow-up Fertil Steril 2010 94 610 618 10.1016/j.fertnstert.2009.01.142 19328465

[B19] Wise LA Rothman KJ Mikkelsen EM Sorensen HT Riis A Hatch EE An internet-based prospective study of body size and time-to-pregnancy Hum Reprod 2010 25 253 264 10.1093/humrep/dep360 19828554 PMC2794667

[B20] Martini AC Tissera A Estofβn D Molina RI Mangeaud A de Cuneo MF Overweight and seminal quality: a study of 794 patients Fertil Steril 2010 94 1739 1743 10.1016/j.fertnstert.2009.11.017 20056217

[B21] MacDonald AA Herbison GP Showell M Farquhar CM The impact of body mass index on semen parameters and reproductive hormones in human males: a systematic review with meta-analysis Hum Reprod Update 2010 16 293 311 10.1093/humupd/dmp047 19889752

[B22] Schneider G Kirschner MA Berkowitz R Ertel NH Increased estrogen production in obese men J Clin Endocrinol Metab 1979 48 633 638 10.1210/jcem-48-4-633 429508

[B23] Giagulli VA Kaufman JM Vermeulen A Pathogenesis of the decreased androgen levels in obese men J Clin Endocrinol Metab 1994 79 997 1000 10.1210/jc.79.4.997 7962311

[B24] Pasquali R Obesity and androgens: facts and perspectives Fertil Steril 2006 85 1319 1340 10.1016/j.fertnstert.2005.10.054 16647374

[B25] Hoffer LJ Beitins IZ Kyung NH Bistrian BR Effects of severe dietary restriction on male reproductive hormones J Clin Endocrinol Metab 1986 62 288 292 10.1210/jcem-62-2-288 3079771

[B26] Leenen R van der KK Seidell JC Deurenberg P Koppeschaar HP Visceral fat accumulation in relation to sex hormones in obese men and women undergoing weight loss therapy J Clin Endocrinol Metab 1994 78 1515 1520 10.1210/jc.78.6.1515 8200956

[B27] Kraemer WJ Volek JS Clark KL Gordon SE Puhl SM Koziris LP Influence of exercise training on physiological and performance changes with weight loss in men Med Sci Sports Exerc 1999 31 1320 1329 10.1097/00005768-199909000-00014 10487375

[B28] Stanik S Dornfeld LP Maxwell MH Viosca SP Korenman SG The effect of weight loss on reproductive hormones in obese men J Clin Endocrinol Metab 1981 53 828 832 10.1210/jcem-53-4-828 7197284

[B29] Strain GW Zumoff B Miller LK Rosner W Levit C Kalin M Effect of massive weight loss on hypothalamic-pituitary-gonadal function in obese men J Clin Endocrinol Metab 1988 66 1019 1023 10.1210/jcem-66-5-1019 3129444

[B30] Pasquali R Casimirri F Melchionda N Fabbri R Capelli M Plate L Weight loss and sex steroid metabolism in massively obese man J Endocrinol Invest 1988 11 205 210 3372960 10.1007/BF03350136

[B31] Bastounis EA Karayiannakis AJ Syrigos K Zbar A Makri GG Alexiou D Sex hormone changes in morbidly obese patients after vertical banded gastroplasty Eur Surg Res 1998 30 43 47 10.1159/000008556 9493693

[B32] Kaukua J Pekkarinen T Sane T Mustajoki P Sex hormones and sexual function in obese men losing weight Obes Res 2003 11 689 694 10.1038/oby.2003.98 12805389

[B33] Niskanen L Laaksonen DE Punnonen K Mustajoki P Kaukua J Rissanen A Changes in sex hormone-binding globulin and testosterone during weight loss and weight maintenance in abdominally obese men with the metabolic syndrome Diabetes Obes Metab 2004 6 208 215 10.1111/j.1462-8902.2004.00335.x 15056129

[B34] Anawalt BD Bebb RA Matsumoto AM Groome NP Illingworth PJ McNeilly AS Serum inhibin B levels reflect Sertoli cell function in normal men and men with testicular dysfunction J Clin Endocrinol Metab 1996 81 3341 3345 10.1210/jc.81.9.3341 8784094

[B35] Jensen TK Andersson AM Hjollund NH Scheike T Kolstad H Giwercman A Inhibin B as a serum marker of spermatogenesis: correlation to differences in sperm concentration and follicle-stimulating hormone levels. A study of 349 Danish men J Clin Endocrinol Metab 1997 82 4059 4063 10.1210/jc.82.12.4059 9398713

[B36] Pierik FH Vreeburg JT Stijnen T de Jong FH Weber RF Serum inhibin B as a marker of spermatogenesis J Clin Endocrinol Metab 1998 83 3110 3114 10.1210/jc.83.9.3110 9745412

[B37] Pierik FH Burdorf A de Jong FH Weber RF Inhibin B: a novel marker of spermatogenesis Ann Med 2003 35 12 20 10.1080/07853890310004084 12693608

[B38] Al-Qahtani A Muttukrishna S Appasamy M Johns J Cranfield M Visser JA Development of a sensitive enzyme immunoassay for anti-Mullerian hormone and the evaluation of potential clinical applications in males and females Clin Endocrinol (Oxf) 2005 63 267 273 10.1111/j.1365-2265.2005.02336.x 16117813

[B39] Muttukrishna S Yussoff H Naidu M Barua J Arambage K Suharjono H Serum anti-Mullerian hormone and inhibin B in disorders of spermatogenesis Fertil Steril 2007 88 516 518 10.1016/j.fertnstert.2006.11.110 17462637

[B40] Goulis DG Iliadou PK Tsametis C Gerou S Tarlatzis BC Bontis IN Serum anti-Mullerian hormone levels differentiate control from subfertile men but not men with different causes of subfertility Gynecol Endocrinol 2008 24 158 160 10.1080/09513590701672314 17926161

[B41] Mabeck LM Jensen MS Toft G Thulstrup M Andersson M Jensen TK Fecundability according to male serum inhibin B--a prospective study among first pregnancy planners Hum Reprod 2005 20 2909 2915 10.1093/humrep/dei141 16024538

[B42] Appasamy M Muttukrishna S Pizzey AR Ozturk O Groome NP Serhal P Relationship between male reproductive hormones, sperm DNA damage and markers of oxidative stress in infertility Reprod Biomed Online 2007 14 159 165 10.1016/S1472-6483(10)60783-3 17298717

[B43] Tuttelmann F Dykstra N Themmen AP Visser JA Nieschlag E Simoni M Anti-Mullerian hormone in men with normal and reduced sperm concentration and men with maldescended testes Fertil Steril 2009 91 1812 1819 10.1016/j.fertnstert.2008.02.118 18423454

[B44] Makler A Zaidise I Brandes JM Elimination of errors induced during a routine human sperm motility analysis Arch Androl 1979 3 201 210 10.3109/01485017908988406 518204

[B45] Menkveld R Stander FS Kotze TJ Kruger TF van Zyl JA The evaluation of morphological characteristics of human spermatozoa according to stricter criteria Hum Reprod 1990 5 586 592 2394790 10.1093/oxfordjournals.humrep.a137150

[B46] Evenson DP Larson KL Jost LK Sperm chromatin structure assay: its clinical use for detecting sperm DNA fragmentation in male infertility and comparisons with other techniques J Androl 2002 23 25 43 11780920 10.1002/j.1939-4640.2002.tb02599.x

[B47] Bungum M Humaidan P Axmon A Spano M Bungum L Erenpreiss J Sperm DNA integrity assessment in prediction of assisted reproduction technology outcome Hum Reprod 2007 22 174 179 16921163 10.1093/humrep/del326

[B48] Maldonado G Greenland S Interpreting model coefficients when the true model form is unknown Epidemiology 1993 4 310 318 10.1097/00001648-199307000-00006 8347741

[B49] Paasch U Grunewald S Kratzsch J Glander HJ Obesity and age affect male fertility potential Fertility and Sterility 2010 94 2898 2901 10.1016/j.fertnstert.2010.06.047 20667533

[B50] HELLER CG CLERMONT Y Spermatogenesis in man: an estimate of its duration Science 1963 140 184 186 10.1126/science.140.3563.184 13953583

[B51] Bonde JP Ernst E Jensen TK Hjollund NH Kolstad H Henriksen TB Relation between semen quality and fertility: a population-based study of 430 first-pregnancy planners Lancet 1998 352 1172 1177 10.1016/S0140-6736(97)10514-1 9777833

